# Neuroinvasion and Viral Reservoir in COVID-19

**DOI:** 10.7759/cureus.11014

**Published:** 2020-10-18

**Authors:** Marcos Altable Pérez, Juan Moises De la Serna

**Affiliations:** 1 Neurology, Virgen De Africa Clinic, Ceuta, ESP; 2 Psychology, International University of Valencia, Valencia, ESP

**Keywords:** covid-19, neuroinvasion, ace2, nicotine, trigeminal, neuroinflammation, central nervous system

## Abstract

The new coronavirus disease 2019 (COVID-19) has a remarkably high transmissibility potential and sometimes invades the central nervous system (CNS). The study of the involvement of the nervous system in the pathogenesis of the disease is especially interesting. Currently, there are only three main theories about it: direct neuroinvasion; blood-brain barrier (BBB) crossing and nicotinic hypothesis. Because of the rapid expansion of a virus that until now was unknown, it is necessary to know the mechanisms by which severe acute respiratory syndrome (SARS)-like coronavirus (SARS-CoV-2) generates the disease. The study of the involvement of the nervous system in the pathogenesis of the disease is especially interesting, since it is the least studied question with more innovative theories that could explain not only neurological complications, but also the primary infection and the involvement of the various organs and systems.

## Introduction and background

Severe acute respiratory syndrome (SARS) is a viral respiratory illness caused by a human coronavirus (HCoV) called SARS-associated coronavirus (SARS-CoV). As occurred in the previous epidemic as a consequence of SARS-CoV-1, currently a new HCoV (SARS-CoV-2) is generating the coronavirus disease 2019 (COVID-19) pandemic. Many other viruses have been described as causing neurological diseases and is the subject of current theories and research. Coronaviruses are causative agents of intestinal, liver, and sometimes neurologic infections [1]. However, the exact infection mechanisms remain unknown, although many are shared among the different types of coronaviruses. The previous SARS epidemic caused by SARS-CoV-1 and its infection mechanisms were widely studied. At present, there are few studies on COVID-19 neuropathogenesis. Furthermore, current reports on neurological symptoms of COVID-19 are being discussed. And reports of virus reactivation in patients who suffered from the disease and who had previously been negative, could also be controversial since it may be the result of faulty tests or, on the other hand, be a falsely negative result in the person who had a positive result in COVID-19. Due to these circumstances, the question is especially interesting nowadays, with multiple reports but few works bringing together the current theories and knowledge.

## Review

SARS is a viral respiratory illness caused by a human coronavirus (HCoV) called SARS-CoV. As occurred in the previous epidemic as a consequence of SARS-CoV-1, currently a new HCoV (SARS-CoV-2) is generating the COVID-19 pandemic. Many other viruses have been described as causing neurological diseases and is the subject of current theories and research. Coronaviruses are causative agents of intestinal, liver, and sometimes neurologic infections [1]. However, the exact infection mechanisms remain unknown, although many are shared among the different types of coronaviruses. The previous SARS epidemic caused by SARS-CoV-1 and its infection mechanisms were widely studied [2]. At present. there are few studies on COVID-19 neuropathogenesis. Furthermore, current reports on neurological symptoms of COVID-19 are being discussed. And reports of virus reactivation in patients who suffered from the disease and who had previously been negative, could also be controversial since it may be the result of faulty tests or, on the other hand, be a falsely negative result in the person who had a positive result in COVID-19. Due to these circumstances, the question is especially interesting nowadays, with multiple reports but few works bringing together the current theories and knowledge.

SARS-CoV-2 or is a new HCoV that emerged in Wuhan, China in late 2019 and is currently causing a pandemic [3]. The way that SARS-CoV-2 enters the brain could be partially responsible for respiratory failure in patients infected with a diagnosis of COVID-19 [4]. The new coronavirus SARS-CoV-2 belongs to a large family of ribonucleic acid (RNA) viruses that has been considered a global health problem since it has a remarkably high transmissibility potential [4] and sometimes invades the central nervous system (CNS) [4].

The CNS is vulnerable to viruses, and many of them end up reaching the brain such as the herpes virus, arboviruses, measles, influenza and HIV, among others. Coronaviruses can also act on the CNS [5-8] and this is why a pandemic with such a high number of affected could mean the appearance of neurological conditions. In fact, 36% of those affected by COVID-19 with neurological manifestations have been reported [9]. Coronaviruses can penetrate the CNS, affect both neurons and glial cells (a property known as neurotropism) and induce various neurological pathologies (neurovirulence) [6]. Regarding the similarity of SARS-CoV-2 with SARS-CoV-1 (causing the epidemic of 2006-2007), it is postulated that SARS-CoV-2 accumulates mainly in the nasal epithelium (neurotropism that would lead to anosmia) and the lower respiratory tract [6]. The appearance of early symptoms in the form of loss of smell (anosmia), imbalance and/or impaired gait (ataxia) and seizures should be considered as neurological manifestations of COVID-19 [7]. Specifically, the coronaviruses have been reported to cross the blood-brain barrier (BBB) [8], and three common virus routes to cross the BBB have been previously reported [9]: direct infection of endothelial cells that comprise the BBB; crossing permeable regions of the BBB and infection of cells that are licensed to cross that barrier, often referred as Trojan horse approach [10]. However, it is unlikely that SARS-CoV-2 can do so, due to the size of this virus, making it more likely to access via olfactory [11] or trigeminal nerves which would explain the anosmia prevalence in this pandemic. The initial mechanism of infection appears to be the recognition by the spike of SARS-CoV-2 of the receptor for angiotensin-converting enzyme 2 (ACE2) in humans, expressed in the capillary endothelium of the brain and other organs [12]. In relation to this information, the presence of SARS-CoV-2 has been recently reported in autopsy samples into CNS level in the endothelial facings, within the areas adjacent to the necrotic areas, in patients with COVID-19 [13]. Furthermore, SARS-CoV-1 has been isolated from brain tissue with neuronal edema and degeneration as seen in autopsies with immunohistochemistry, in situ hybridization, and electron microscopic confirmation of viral infection of neurons [14].

However, it can be considered that there are currently two forms of CNS involvement that are not mutually exclusive: the theory of direct neuroinvasion (hypothesis of synaptic propagation and hypothesis of ACE2) and the nicotinic hypothesis.

1. Direct neuroinvasion hypothesis

The hypothesis about neuroinvasion and neurovirulence properties of SARS-CoV-2 is based on the following pieces of evidence: extrapolated biological plausibility of CNS involvement by other respiratory viruses; evidence of neurological damage by coronavirus in other species; animal models of CNS infection by HCoV; the existence of neurological complications from other coronaviruses; COVID-19 patients who have presented neurological manifestations [15].

The first description comes from He et al. [4]. COVID-19 patients have respiratory distress and are sometimes unable to breathe spontaneously. Additionally, they may show neurological signs, such as headache, nausea, and vomiting. Growing evidence shows that coronaviruses are not always limited to the respiratory tract but can also invade CNS and cause neurological disease. In fact, SARS-CoV-1, the most closely related HCoV, has been seen in the brains of patients and experimental animals, where the brain stem was severely infected [8], and SARS-CoV-2 has been identified in the cerebral spinal fluid (CSF) [16].

Previous studies have demonstrated the ability of this virus to cause neuronal death in mice through the invasion of the CNS by the cribriform plate of ethmoid and subsequent invasion of the olfactory neuroepithelium [17]. Coronaviruses could spread through synapses from the neurons of the olfactory nerve to the cardiorespiratory centre and from there reach the lungs through the medulla, ending in the afferent neurons located in the lung for their respiratory control (theory of synaptic propagation). This also further suggests that COVID-19 neurotropism may contribute to respiratory failure. When the virus enters the body through the nerves and/or the lung, it would lead to different clinical characteristics with different results, as reported. This pathway has been described in many viruses, and even prions, through the peripheral nervous system [11]. SARS-CoV-2 could directly penetrate sensory nerve endings and travel through retrograde axonal flow as other coronaviruses [18]. Here it is worth noting the role of the trigeminal nerve at the entrance to the CNS since cases of conjunctivitis have been reported [19], and asymptomatic presence of SARS-CoV-2 on the ocular surface only has been reported as an exit route. Another route of neuroinvasion less probably is the via described in prions [20]. The prions accumulate in sympathetic nerve endings within lymphoid organs, which are a reservoir of infectivity. Through sympathetic nerves, prions spread to the CNS where they replicate in neurons, causing their destruction in encephalopathies [21]. Direct neuroinvasion contrasts with the currently accepted view that ACE2 is the primary receptor for SARS-CoV-2 for its entry into cells [11].

A second line of argument that underlines the neuroinvasion hypothesis comes from a study by Baig et al. [12]. In the short time after the outbreak, it has been shown similar to SARS-CoV-1, SARS-CoV-2 exploit the ACE2 receptor to penetrate into the cells. In the brain, ACE2 is expressed in neurons, glia, and endothelial cells, and is particularly present in the brainstem and in the regions responsible for the regulation of cardiovascular functions, including the subfornical organ, paraventricular nucleus, nucleus of the solitary tract, and rostral ventrolateral medulla [22]. Once inside the neuronal tissue environment, SARS-CoV-2 interaction with ACE2 receptors expressed in neurons can initiate a cycle of viral gemmation accompanied by neuronal damage without substantial inflammation, as seen with SARS CoV-1 in the past [18]. This would explain the lightness of the symptoms in a great number of cases of COVID-19. Several studies have reported that some patients with COVID-19 show neurological symptoms such as headache (about 8%), nausea, and vomiting (1%) [23]. However, cases of SARS-Cov-2 encephalitis and its presence in cerebrospinal fluid have already been recorded [16,23,24]. Regarding endothelial cell involvement, emphasis has recently been placed on cytokine storm and neuroinflammation [25]. Cytokine storm, also called macrophage activation syndrome, is a systemic inflammatory response that can be triggered by a variety of factors such as infections and drugs [26].

2. Nicotinic hypothesis

The relationship between nicotine and ACE2 has been explored in the framework of cardiovascular and lung diseases [27]. Angiotensin-(1-7) or ANG (1-7) is a heptapeptide of the renin angiotensin system (SRA) generated from angiotensin 2 (ANG 2) and angiotensin 1 (ANG 1) through ACE2, among other enzymatic routes. ANG (1-7) binds to the G protein-coupled metabotropic receptor MAS [28]. It is considered a physiological antagonist of ANG 2, so it is postulated that ANG (1-7) and other MAS agonists could reduce various deleterious actions of ANG 2 in the brain and other body organs [29]. In the ACE-angiotensin 2 (ANG 2)-angiotensin receptor type 1 (AT1R) system, nicotine increases the expression and activity of renin, ACE and AT1R, while in the compensatory axis ACE2/ANG- (1- 7)/MAS, nicotine downregulates the expression and/or activity of ACE2 and AT2R, suggesting a possible contribution of nicotinic acetylcholine receptors (nAChR) in the regulation of ACE2. Although a lower incidence of COVID-19 has been described in smokers [30], this possibility has not yet been explored in the framework of viral neuroinfections [30]. Furthermore, it could be a therapeutic target in SARS-CoV-2 infection.

From another point of view, various coronaviruses have been identified by serological techniques in a wide variety of neurological pathologies, such as Parkinson's disease, amyotrophic lateral sclerosis, multiple sclerosis, and optic neuritis [7,19,31,32]. Viral persistence of Nidovirus (coronavirus) in CNS has been described by Lavi et al. in 1999 [33]. Coronaviruses 229E, 293, and OC43 have been isolated from the cerebrospinal fluid and the brain of patients with multiple sclerosis [31]. The immune response after infection could participate in the induction or exacerbation of multiple sclerosis outbreaks in susceptible individuals [5]. This would also support the idea that apparent reinfections of COVID-19 patients who passed the disease, return to suffer it with or without neurological symptoms, due to the persistence of the virus in neural tissue. This has been described in varicella-zoster virus infection [34,35]. In COVID-19, findings from a small group of cases suggested that there was currently evidence for reactivation of SARS-CoV-2, has no specific clinical characteristics to distinguish from primoinfection [36].

In addition, other viruses, such as the herpesviruses including the herpes simplex virus and the herpes zoster virus, among others, reach from the nerve endings to the spinal ganglia and remain in a state of latency with low replication, until a reactivation due to a disturbance of the immune system [37]. Nipah virus (a paramyxovirus) causes many of the same respiratory and neurological symptoms that SARS-CoV-2. Kevin R in 2020 [38] showed a reactivation, months or years later of the Nipah virus from latent infections. Both viruses, (SARS-CoV-2 and Nipah virus) selectively infect neurons, and this enables them to evade attacks from the immune system of the host. This occurs because, as is known, almost all T‐cell (lymphocytes) activations require that an antigen be presented by another cell, such as a dendritic cell, on a specific surface protein known as a major histocompatibility complex (MHC) or human‐leukocyte-associated (HLA) protein in humans [39]. Lymphocytes with the MHC requirement for antigen presentation are activated. MHC class II for presentation to cluster of differentiation (CD)4 T cells and MHC class I for presentation to cytotoxic CD8 T cells [39]. But neurons carry very few of these MHC proteins, so they cannot easily present viruses on MHC class I to cytotoxic CD8 T cells to induce an attack on the infected neurons [37].

## Conclusions

The mechanisms by which SARS-CoV-2 reaches the CNS and produces different neurological manifestations, remains unknown. Currently, there are two main ways proposed that are not exclusive to each other. On the one hand, through neuroinvasion, either through the BBB, either through the peripheral nerves or its interaction with ACE2 receptor. And, on the other hand, the modulation of the renin-angiotensin system by nicotinic acetylcholine receptors and the deleterious effects of ANG 2 on the brain and other organs. The study of the involvement of the nervous system in the pathogenesis of COVID-19 is especially interesting since it is the least studied question with more innovative theories that could explain not only neurological complications but also the primary infection and the involvement of the various organs and systems. More studies are needed to clarify the neuropathogenesis of COVID-19.

## References

[1] Guan WJ, Ni ZY, Hu Y (2020). Clinical characteristics of coronavirus disease 2019 in China. N Engl J Med.

[2] Ding Y, He L, Zhang Q (2004). Organ distribution of severe acute respiratory syndrome (SARS) associated coronavirus (SARS-CoV) in SARS patients: implications for pathogenesis and virus transmission pathways. J Pathol.

[3] WMH Commission (2020). WHO: pneumonia of unknown cause - China. https://www.who.int/csr/don/05-january-2020-pneumonia-of-unkown-cause-china/en/.

[4] He F, Deng Y, Li W (2020). Coronavirus disease 2019: What we know?. J Med Virol.

[5] Chen J (2020). Pathogenicity and transmissibility of 2019-nCoV—a quick overview and comparison with other emerging viruses. Microbes Infect.

[6] Desforges M, Le Coupanec A, Dubeau PM, Bourgouin A, Lajoie L, Dube M, Talbot PJ (2020). Human coronaviruses and other respiratory viruses: underestimated opportunistic pathogens of the central nervous system?. Viruses.

[7] Mao L, Jin H, Wang M (2020). Neurologic manifestations of hospitalized patients with coronavirus disease 2019 in Wuhan, China. JAMA Neurol.

[8] Baig AM (2020). Neurological manifestations in COVID-19 caused by SARS-CoV-2. CNS Neurosci Ther.

[9] Joob B, Wiwanitkit V (2015). Neurologic syndrome due to MERS: is there a possibility that the virus can cross the blood-brain barrier to cause a neurological problem?. Ann Trop Med Public Health.

[10] Miller KD, Schnell MJ, Rall GF (2016). Keeping it in check: chronic viral infection and antiviral immunity in the brain. Nat Rev Neurosci.

[11] Kim KS (2008). Mechanisms of microbial traversal of the blood-brain barrier. Nat Rev Microbiol.

[12] Baig AM, Khan NA (2014). Novel chemotherapeutic strategies in the management of primary amoebic meningoencephalitis due to Naegleria fowleri. CNS Neurosci Ther.

[13] Baig AM, Khaleeq A, Ali U, Syeda H (2020). Evidence of the COVID-19 virus targeting the CNS: tissue distribution, host-virus interaction, and proposed neurotropic mechanisms. ACS Chem Neurosci.

[14] Poyiadji N, Shahin G, Noujaim D, Stone M, Patel S, Griffith B (2020). COVID 19-associated: acute hemorrhagic necrotizing encephalopathy: imaging features. Radiology.

[15] Gu J, Korteweg C (2007). Pathology and pathogenesis of severe acute respiratory syndrome. Am J Pathol.

[16] Carod Artal FJ (2020). Complicaciones neurológicas por coronavirus y COVID-19 [Article in Spanish]. Rev Neurol.

[17] Holshue ML, DeBolt C, Lindquist S (2020). First case of 2019 novel coronavirus in the United States. N Engl J Med.

[18] Netland J, Meyerholz DK, Moore S, Cassell M, Perlman S (2008). Severe acute respiratory syndrome coronavirus infection causes neuronal death in the absence of encephalitis in mice transgenic for human ACE2. J Virol.

[19] Shindler KS, Chatterjee D, Biswas K (2011). Macrophage-mediated optic neuritis induced by retrograde axonal transport of spike gene recombinant mouse hepatitis virus. J Neuropathol Exp Neurol.

[20] Xie HT, Jiang SY, Xu KK, Liu X, Xu B, Wang L, Zhang MC (2020). SARS-CoV-2 in the ocular surface of COVID-19 patients. Eye Vis.

[21] Glatzel M, Heppner FL, Albers KM, Aguzzi A (2001). Sympathetic innervation of lymphoreticular organs is rate limiting for prion neuroinvasion. Neuron.

[22] Matías-Guiu J, Gomez-Pinedo U, Montero-Escribano P, Gomez-Iglesias P, Porta-Etessam J, Matias-Guiu JA (2020). Should we expect neurological symptoms in the SARS-CoV-2 epidemic? [Article in Spanish]. Neurologia.

[23] Li YC, Bai WZ, Hashikawa T (2020). The neuroinvasive potential of SARS-CoV2 may be at least partially responsible for the respiratory failure of COVID-19 patients. J Med Virol.

[24] Wu Y, Xu X, Chen Z (2020). Nervous system involvement after infection with COVID-19 and other coronaviruses. Brain Behav Immun.

[25] Serrano-Castro PJ, Estivill-Torrúsbc G, Cabezudo-García P (2020). Impact of SARS-CoV-2 infection on neurodegenerative and neuropsychiatric diseases: a delayed pandemic? [Article in Spanish]. Neurología.

[26] Shimabukuro-Vornhagen A, Gödel P, Subklewe M (2018). Cytokine release syndrome. J Immunother Cancer.

[27] Oakes JM, Fuchs RM, Gardner JD, Lazartigues E, Yue X (2018). Nicotine and the renin-angiotensin system. Am J Physiol Regul Integr Comp Physiol.

[28] Souza-Santos RA, Sampaio WO, Alzamora AC, Motta-Santos D, Alenina N, Bader M, Campagnole-Santos MJ (2018). The ACE2/angiotensin-(1-7)/Mas axis of the renin-angiotensin system: focus on angiotensin-(1-7). Physiol Rev.

[29] Miyara M, Tubach F, Pourcher V (2020). Low incidence of daily active tobacco smoking in patients with symptomatic COVID-19. Qeios.

[30] Changeux JP, Amoura Z, Rey F, Miyara M (2020). A nicotinic hypothesis for Covid-19 with preventive and therapeutic implications. Qeios.

[31] Shang J, Ye G, Shi K (2020). Structural basis of receptor recognition by SARS-CoV-2. Nature.

[32] Burks JS, DeVald BL, Jankovsky LD, Gerdes JC (1980). Two coronaviruses isolated from central nervous system tissue of two multiple sclerosis patients. Science.

[33] Lavi E, Schwartz T, Jin YP, Fu L (1999). Nidovirus infections: experimental model systems of human neurologic diseases. J Neuropathol Exp Neurol.

[34] Fields BN, Knipe DM, Howley PM (1995). Fundamental Virology, Third Edition.

[35] Gershon AA, Gershon MD (2013). Pathogenesis and current approaches to control of varicella-zoster virus infections. Clin Microbiol Rev.

[36] Ye G, Pan Z, Pan Y (2020). Clinical characteristics of severe acute respiratory syndrome coronavirus 2 reactivation. J Infect.

[37] Murphy K (2012). Failures of host defense mechanisms. Janeway's Immunobiology, 8th Edition.

[38] Kevin R (2020). Explanation for COVID-19 infection neurological damage and reactivations. Transbound Emerg Dis.

[39] Alberts B, Johnson A, Lewis J (2002). Chapter 24: the adaptive immune system. Molecular Biology of the Cell, 4th Edition.

